# Tū Kaha: He mōhio ki ngā Māori o te kōmaoa waewae (Stand Strong: A qualitative study of Māori with venous leg ulcers in Aotearoa New Zealand)

**DOI:** 10.1177/13591053241289049

**Published:** 2024-10-18

**Authors:** Jacquie Kidd (Ngāpuhi), Mary-Kaye Wharakura (Tainui / Ngāpuhi), George Laking (Te Whakatōhea), Dianna McGregor (Ngāpuhi), Rosie Dobson, Andrew Jull

**Affiliations:** 1 Auckland University of Technology, New Zealand; 2 University of Auckland, New Zealand; 3 Te Whatu Ora - Waitematā, New Zealand

**Keywords:** experience, Indigenous population, Māori, New Zealand, qualitative research, venous leg ulcers

## Abstract

Venous leg ulcers have impact on people’s lives far beyond that of a skin lesion but these impacts have not been explored from an Indigenous perspective. We used a Māori-centered narrative approach to interview 13 Māori in Aotearoa New Zealand with venous leg ulcers. Data analysis was informed by a reflexive thematic approach and four themes were identified: Ko waewae ahau (I wear the leg); Ngā mea hōhā (annoying things); Ka tangi te ngākau (heartfelt grief); and Mamae (pain, sore, hurt). Recognizing patients’ expertise in their condition, the inclusion of whānau (family) in care planning and provision, while providing consistent advice and resource access, would all enhance the experience of venous ulcer management. Training in venous leg ulcer care needs to move beyond a focus on the leg and toward a more holistic approach that encompasses a broader understanding of patient experiences and cultural contexts when managing venous ulcers.

## Introduction

Leg ulcers are non-healing lesions of the lower leg and between 50% and 70% of all leg ulcers in the developed world are venous leg ulcers (Baker et al., 1992; Nelzen, 2007). Venous leg ulcers (VLU) are the most severe presentation of chronic venous insufficiency and venous hypertension and are characterized by remitting and relapsing shallow moist wounds, leg edema, venous eczema, and trophic skin changes such as hemosiderin pigmentation. The prevalence of VLU is about 3.2/1000 people (Probst et al., 2023), but prevalence increases with age (Nelzen, 2007). However, age of onset may be moderated by ethnicity as recent reports in Aotearoa New Zealand suggest that VLU occur in Māori at a younger age than non-Māori and non-Pasifika populations (Blackmore et al., 2022; Bourke and Scott, 2021). While health-related quality of life is significantly lower across the board in people with venous leg ulcers compared to age and sex-standardized population norms (Jull et al., 2018), again age moderates these effects with health-related quality of life being more affected in those aged younger than 65.

Typically, treatment of open VLU involves long periods in high compression bandages or hosiery, with regular contact with a nurse for dressing changes and/or application of new bandages. A systematic review of qualitative studies found open VLU created considerable burden on the sufferers’ lives (Phillips et al., 2018). Once healed, preventing further VLU requires wearing high compression hosiery (Nelson and Bell-Syer, 2014), but healing loses its positive connotations and is seen as a temporary victory in a process of forever healing (Chase et al., 1997). Such perceptions are also reflected in feelings of powerlessness with concerns about the ulcer dominating peoples’ lives and recurrence being perceived as inescapable. That said, people desperately wanted their ulcers to heal (Bland, 1994). However, none of these studies reported on the experiences of an Indigenous population. Indigenous populations conceptualize health in broader terms to that of prevalent Western medical models (Durie, 2003), and a Māori model defines health in terms of balance in the realms of the physical, spiritual, wider family, the mind, and the wider environment (Durie, 1985). Given this lack, we aimed to explore the experience of whānau Māori (Māori families).

## Methods

We used a decolonizing and a Māori-centered approach to conducting and reporting this study. Such an approach is about making Indigenous concerns and worldviews central to our research method and theorizing (Tuhiwai Smith, 2021). To ensure such an approach, the research team comprised a majority of Māori researchers to guarantee Māori governance, the project employed a Kairangahau Māori (Māori researcher) as the key person to engage with participants, we followed tikanga Māori (customary Māori practices) in the conduct of the research, and we prioritized the use of the Indigenous language for key terms and concepts throughout the report followed by English (the colonizers’ language) in parentheses. In this report, once the kupu Māori (Māori words) have been introduced in te reo Māori (the Māori language), they are not translated thereafter. We have not italicized terms in te reo Māori, as that can be conceived as a form of othering the language and those who use it. However, all quotations are provided in italics and where te reo Māori occurs within a quotation it is presented congruent with the adjacent text.

We recruited participants from people who took part in two randomized controlled trials of interventions for treating VLU conducted in Aotearoa New Zealand between 2015 and 2018 (Jull et al., 2017, 2020), using a convenience sampling approach. All participants in the trials had completed a question about self-identified ethnicity for the baseline data collection. Sixty-two people from the two trials identified as Māori, with most living in the Auckland and Waikato regions. We cross-tabulated these people with responses to a question on participating in future research. The remaining people were then approached by the Kairangahau Māori. If they expressed interest, we asked them to discuss participation with the whānau (family) and invite whānau to participate in a hui (meeting) with the Kairangahau. A combined participant information sheet and consent form was mailed to the whānau and discussed at the first visit. No information at the visit was collected until each of the whānau members provided written consent. Following written consent, the interviews were recorded on a computer tablet. We aimed to interview between 10 and 20 participating whānau. Participants were recruited and interviewed between November 2019 and June 2020.

Data analysis followed a wānanga process (thought space), a process of deliberation that was informed by reflexive thematic analysis (Braun and Clarke, 2022). In the first instance the narratives were transcribed by MKW and checked with whānau for accuracy and to ensure that their stories were the ones they intended to share. The narratives were anonymized using a pseudonym and the finalized transcripts were shared with the wider research rōpū (team), who engaged in an initial individual process of close reading and identifying codes. The rōpū then gathered for a day to wānanga; we used large sheets of paper taped to the wall to which we added pseudonyms and some demographic detail. We then shared our thoughts about the main points or codes from each narrative with the supporting quotes. We gave our full attention to one whānau at a time, before we considered all the data. Once each whānau was represented in our space, we began looking at themes that occurred across the stories. We were able to walk around to “visit” each narrative, touch the paper, take breaks for kai (food), and share our whakaaro (thoughts) through te reo Māori, colloquial and academic language, linguistic code switching, humor, and metaphor. Those initial themes were, through a process of analysis that was firmly located in Te Ao Māori (the Māori worldview), gradually refined by the rōpū into a final set of four. The elucidation of the themes with relevant quotes was delegated to three of the rōpū given their (1) engagement with the participating whānau (MKW), (2) clinical and research expertise in VLU (AJ), and (3) expertise in qualitative research and mātauranga Māori (JK). The quotes were referenced using the participants’ pseudonyms and tribal affiliation. Approval for the study was obtained from the Waikato District Health Board Te Puna Oranga Māori Consultation Research Review Committee and the Auckland Health Research Ethics Committee on behalf of the Auckland and Counties Manukau District Health Boards (#000143).

## Results

Thirteen participants with a VLU were interviewed; eight participants were interviewed prior to the SARS-CoV-2 contact restrictions from 23 March 2020, three were interviewed via Zoom during the contact controls, and the remaining participants were interviewed after the restrictions were relaxed in early May 2020. The participants’ median age was 61 years (range 39–87), eight were wāhine (women) and five were tāne (men). Seven had been participants in Keratin4VLU and six in Aspirin4VLU. Iwi (tribal affiliations) were identified by 12 of the 13 participants. Two whānau members were included, one participating in the interview with her mātua (parent) and the other, a former hoa wahine (female partner) interviewed separately at the participant’s request. Pūrākau (stories) shared with us generated four themes: ko waewae ahau (I wear the leg), ngā mea hōhā (annoying things), ka tangi te ngākau (heartfelt grief), and mamae (pain). In this paper we use the Māori language term “whanau” interchangeably to refer to “participants” or “patients.”

### Ko waewae ahau, “I wear the leg”

Whānau living with a VLU often spend years engaged with the ulcer, trying to heal it. The ulcer can become the focus of all attention, and in being central to how people then live their lives, it can be perceived as controlling the person— “*my life’s around my leg*”*(Roera, Ngāti Whātua)* leading to hypervigilance about their leg.

It just takes a long time getting out of the car, ‘cause you’ve gotta be very careful … I’ve gotta be very careful on how I don’t walk into things with my legs, my skin is so fragile now. (Timoti, Ngāti Porou)

Although the everyday lives of whānau revolved around their legs, they still displayed remarkable manawanuitanga (patience, effort) in getting on with life— “*you know I could deal with it, this problem. I just coped*”*(Hemi, Ngāpuhi)*, even to the point of not showing the effects on their lives.

He would rather wear the taumaha (weight, burden) himself, than put it on anyone else. (Mana, Ngāti Whātua)

However, having VLU often requires regular visits with district nurses and less frequent visits with other professionals, with routines constructed around these visits; such routines might consist of removing bandages and dressings, bathing or showering the leg before a district nurse arrives or routines that involve total self-management of the VLU. Consequent to this focus and these routines, people develop a heightened body awareness, an expertise in the condition of and changes in their own bodies.

You absolutely get to know your body after a while. You do know your body. (Roimata, Ngāpuhi)

The participants’ literacy in their own bodies extends from obvious changes in the color of skin *(Timoti)* to noticeable differences in the leg and ulcer during menstruation *(Anahera)* to knowledge of the subtle sensations associated with the recurrence of an ulcer.

Soon as I think I was going to get an ulcer, had a little sign of an ulcer … I call the GP and he sends a referral to the nurse. I did it last time and they were out here the next day … I still get a tingling like a bit of a sting or something sometimes and that’s when I say to her [daughter] to have a look to make sure it’s not coming back … (Heeni, Tainui)

Health care professionals can enable the whānau to self-manage their condition through acknowledging and honoring the patient’s knowledge and expertise.

… my regulars are actually listening to me, they say ‘oh yes because you’re the best person that would know’ … they give me all the dressings and let me do it and I watch it and if don’t feel I need to have a cover dressing on, I can just cover it with nothing so I can just let it breathe for a while. (Roera, Ngāti Whātua)

However, health professionals can also act as impediments to the whānau acting on their own knowledge and controlling in their own care. Those impediments may be as insidious as unwittingly sowing doubt in the person’s own knowledge or as overt as ignoring the person’s wishes.

Then you get some that come in … where they feel they know better than you. So they give you little bit of doubt … and make you think “oh maybe they are right and maybe I should” ’ even though you know it’s not. (Roera, Ngāti Whātua)

You know sometimes they would push one [dressing] I didn’t like and I’d say nah. And if they put it on, I’d take it off. (Heeni, Tainui)

Health care professionals ignoring the right of whānau to rangatiratanga (exercise of full authority) over their own body is ignoring their autonomy, arousing anger as the person’s knowledge, strengths, and rights are subjugated by the health care professional. Having to stand up for their rangatiratanga is exhausting, spiritually, mentally, culturally, and physically, impacting the wellness of the whānau. Māori perceive this as an assault that tramples on the mana (prestige or authority) of the individual, the whānau, their whenua (land) and of Maori as a collective people.

Maybe they should listen to the one who’s wearing the ulcer … they overpower us with their stories and they don’t want to listen to our story because they think ‘what the hell do you know?’ But we wear the leg, we know everything. (Kepa, Tainui)

So they’re only starting to listen to me now because I know what works for my leg but yeah it’s really exhausting. (Anahera, Ngāti Maniapoto)

The expression of rangatiratanga can be upheld by the wider whānau who can play a substantial role in supporting the person and in providing meaningful support and care for that person’s struggles. The nature of a long term wound and the restrictions it can create means the VLU can be depleting of the person’s mana (inherent worth) and wairua (spirit). Having the aroha (love, respect) and support of whānau by their side nurtures the mana of the person and whānau, building strength and dignity.

I’m thankful, like, for my wife and my kids and my mokopuna, you know, they’re the ones that keep me going. You know, without them - I’m not gonna say it’s a waste of time living, you know. But without them, I wouldn’t be where I am today. (Timoti, Ngāti Porou)

### Ngā mea hōhā, annoying things

Inconsistency in care was featured in the list of ngā mea hōhā; participants repeatedly mentioned nurses having favored dressings or different approaches to ulcer management, which often left the participant feeling the subject of experimentation or simply resigned to the fact that each nurse had a different approach.

I had that ulcer for five or six years. It was during that period where they were experimenting. Every different nurse had a different way of trying to heal it … (Heeni, Tainui)

I have so many nurses that have different ways. They like to try it their way, you know. So I let them try it their way and that’s why my leg doesn’t heal … (Kepa, Tainui)

Nurses would still change the management plan despite explicit written instructions to not do so from a health care practitioner whom the person wearing the leg trusts.

And I’ve had one for a long time who has stipulated on my form DO NOT CHANGE … and that’s exactly what happened. Someone new came along and changed and it [the venous leg ulcer] flared back up again. (Roera, Ngāti Whātua)

The involvement of many different nurses also influenced the quality of relationship between the whānau and the nursing team, and there is a toll on the patient as they learn to adapt to each new nurse. Expectations and trust that developed as a nurse works with a patient can then be profoundly disturbed by the introduction of a new nurse.

It’s quite traumatising going from one nurse to another nurse. There’s quite a big ordeal. They don’t realise how much the patient needs them. It’s quite an ordeal. (Hera, Tainui)

Whānau experiences with advice from different disciplines can also lead to confusion in whom to trust and whose advice to follow.

The doctor told me not to sit down too long and the nurse said lie down and I don’t know whose advice to take. Don’t sit down too long, you might die, if you don’t lay down, you’ll die. (Tipene, Tainui)

One whānau suggested that nurses could mitigate some of this frustration by showing manaakitanga (hospitality, kindness) and demonstrating that they were interested in working with the whānau.

[Just check] how’s the whānau, how are they going, how are you doing? And … did you know of services in the community that you can talk to, or services that can help the whānau? Just a simple thing like that would be enough to let the whānau know, oh this is a caring person, now this person is asking the right questions. Because this person understands it and somebody else cares. (Mana, Ngāti Whātua)

The whānau described many practical barriers to receiving good care. The costs of care to whānau extended beyond the district nursing care, creating added burden for those least able to afford it.

The pick-ups, travel, car parks and the care, that’s what I’m going through now (Awhina’s daughter, Ngāti Whātua)

Furthermore, whānau reported inter-regional variation in the support they received to prevent venous leg ulcers. Compression hosiery (stockings) reduce the risk of VLU recurrence and need to be worn daily once an ulcer heals. Whānau reported having these provided to them free (although not initially told that could be the case) as well as having to take on the cost themselves.

When I first got here to [named town] and then no-one told me I was allowed a free pair … until it just happened … and they say to me you’re allowed to have two pairs of hosiery socks a year. (Timoti, Ngāti Porou)

I’ve spent thousands over ten years, $83 say every three months but I have to have them, they [stockings] were a great thing for me. (Tipene, Tainui)

Another barrier to good care is the inadequacy of resourcing for the nursing services and impractical solutions that can arise from that inadequacy. Whānau reported that the range of dressings available could be poor or that provision of hosiery did not account for their actual needs.

They ‘make do’ with what they have at the clinic. They have rubbish plasters that stick to your leg and expect it to hold an inner bandage - on a wet wound. The nurses do their best but it’s all they got. You can’t get the other stuff so they only do or use what they got. (Awhina, Ngāti Whātua)

I said that’s absolutely ridiculous [providing only one pair of stockings], what did they think you needed to do with the one pair that you got? You’ve got to wash it everyday now. (Mana, Ngāti Whātua)

I want those dressings at the homes of the people, rather than at the heart of the bloody hospital board. (Taika, Ngāpuhi)

The inadequacies in resourcing extended beyond those associated with direct care of the venous leg ulcers, but the siloed service provision meant there was little understanding of or support for the needs of whānau caregivers and no understanding of the disability that venous leg ulcers can create.

She’s [his daughter] given up her job. Yeah so she can come down here and look after me. She wanted the Government or WINZ (Work and Income New Zealand, the government social welfare agency in Aotearoa New Zealand) to give her a job looking after me but they won’t aye? (Tipene, Tainui)

What support for us as a caregiver, what support is available to us? … [Social] housing won’t build me a ramp, so I’ve got to climb these steps and it’s so sore. (Awhina, Ngāti Whātua)

The importance of addressing systemic resource deficiencies and providing adequate support at both the individual and community levels reflects the need for a collective responsibility; whānau, community, and government to ensure the wellbeing of those “who wear the leg” and their caregivers within the Māori community.

Whānau also expressed strain and distress with other aspects of having a VLU that might be considered minor but had a lasting impact on the ways Māori with VLUs lived their lives or expressed their identity. Participants detailed not being able to wear shorts, having difficulty with wearing shoes because of the bulkiness of bandages, appearing nice for special occasions, and were troubled by having to wear a stocking if male or not being able to shave their leg if female.

I couldn’t wear shorts ‘cause I was a bit shy, a bit whakamā (strong feelings of shame) in doing it. (Timoti, Ngāti Porou)

I wanted to go to a, say like a wedding or somewhere you had to dress up. What do you wear on your feet, sandals? … you really can’t adjust the clothing because of the big leg, with the big bandaging, and the other one wasn’t [big], so really had to think about what I could wear, make myself look tidy and respectable. (Roera, Ngāti Whātua)

There is whakamā around it you know … just wearing the stockings, the bandages and all that means … (Ropata, Whakatōhea)

On the leg that is affected … I can’t shave my leg down there. (Tui, Tainui)

It was notable that whānau expressed all these quite significant concerns as irritations or frustrations. However, as highlighted in the next theme, there is also a deep grief associated with having VLU for the participants.

### Ka tangi te ngākau, heartfelt grief

While some participants relied on whānau support, other participants’ response was to isolate themselves from whānau and friends in the hope that doing so will help reduce worry and protect their whānau from the burden of the participant’s suffering.

I try to be [relaxed] with my whānau and just tell them everything is okay ‘cos I don’t want them worrying about it, yeah. So I just say ‘oh all good’, even if it’s not. (Anahera, Ngāti Maniapoto)

This distancing from their physical, emotional, spiritual, and cultural supports comes from the position of aroha and manaakitanga. However, there is also recognition of the consequences for their whānau, who lack knowledge of the true impact of ulcers, of not being able to nurture and support their loved member of the whānau, causing the pain of mokemoke (loneliness) from estrangement within the whānau.

It hurts them because I don’t let them close, so it hurts in that way. And not telling them hurts them as well. [Hera, Tainui]

For some, the desire to protect meant that those with the ulcers also sacrificed all intimate relationships:

Relationships, that was another ‘don’t go there’, don’t start a problem for someone else … I told my partner ‘you want to split, go for it, I think this is going to stuff me up’ … I didn’t want to hang out with girls and put my sickness and plant it on them – no. I didn’t have a relationship because of that and plant my problems onto somebody else (Hemi, Ngāpuhi)

Having an open wound can cause embarrassment for the smell and associated messiness, issues which evoke whakamā, leading to avoidance of social contacts and relationships not enduring.

You feel embarrassed and you go and shy away from people. Friends, family. And when it’s the most hot summer day, they all wanna go to the beach and of course you wanna go but you hold back because you feel embarrassed. And I have a big family and there’s lots of outings and I hold back from nearly all of them. (Hera, Tainui)

Important familial relationships, for instance with mokopuna (grandchildren), were also not sustained, with the opportunities for intergenerational relationship and exchange being disrupted, opportunities that are essential for the transmission of mātauranga (knowledge) between the old and young.

The ulcers alienated my mother from her friends. It alienated my mother from her family. A lot of the kids wouldn’t come around and visit her either because Mum stayed in the bedroom the whole time. Her leg was always open, the ulcer was always open (Taika, Ngāpuhi)

The participants also noted that their opportunity for full participation in Te Ao Māori (the Māori world) was truncated. Participation in ceremonies that are components of a full cultural life, such as pōwhiri (rituals of welcome) and tangihanga (rituals of funerary), were restricted. Tangihanga are among the most important institutions in Māori life, especially for those in leadership roles, with whaikōrero (oratory), waiata (song), and ceremony over several days celebrating and farewelling the departed, while supporting and renewing bonds with those who remain. Limited participation restricts the opportunity for that renewal and the resolution of grief.

I go to tangi (funerary rituals). I’d just go there, pay my respects and then go. Can’t stay with the leg, no. (Ropata, Whakatōhea)

The inability to participate fully in Māori customs and traditions due to the VLU underscores the profound impact of physical limitations on the ability to fulfill important cultural roles and responsibilities.

### Mamae, pain

The issue of pain associated with VLU was central to all the pūrākau, as expected.

I used to get the worst pain. My whole body was yeah … throbbing. It was just like that painful … I was curled up like a foetus and squeeze my leg together because it was sore … And it was wicked, wicked, wicked. (Hemi, Ngāpuhi)

Managing the pain takes time and effort for the person and their whānau, further exacerbating the wide-ranging impact of this condition.

When my leg gets tired my whole leg starts to drop and gets heavy. I try and give myself 15 minutes here and there throughout the day and keep up with paracetamol with codeine, and that’s for the pain (Hera, Tainui)

However, many whānau described continuing to maintain their daily activities while they were in pain, showing a determination to resist the impact of their ulcers.

I never let them stop me from doing anything. I was very sore but I didn’t care, I just work, work, work (Awhina, Ngāti Whātua)

My pain also you know, I have this and I think it’s Māori that do this, as well. We just have this thing of how we see, how we present ourselves to the whānau and how we see the whānau. I don’t want to show that weakness in front of my whānau. (Ropata, Whakatōhea)

In the context of Te Ao Māori, the pain from ulcers was a private matter. This reflects a cultural value of self-sufficiency and resilience within Māori communities, where the tendency is to not burden others with one’s suffering, prioritizing the collective well-being and dignity of whānau.

## Discussion

The four themes in our study have highlighted different ways whānau have experienced the impact of VLU on their lives. From the expert position of “wearing the leg” (ko waewae ahau), through the irritations associated the condition (ngā mea hōhā), to the grief of interrupted relationships (ka tangi te ngākau), and persistent physical pain (mamae), the whānau have described a cumulative effect that changes lives at all levels. These themes highlight the exhausting burden associated with a VLU, with a participant summarizing “*I’ve had enough. I feel like it’s holding me back … Why are years wasted worrying about a stupid bloody leg?*”*(Anahera, Ngāti Maniapoto).*

Broadly, the four themes are also reflected in recent review of thirteen qualitative studies that found the effects of VLU could be grouped under physical impacts, psychological impacts, social impacts, and treatment impacts (Phillips et al., 2018). The physical impacts were related to pain, odor, excessive exudate, itching, and limitations on mobility, which then affect activities of daily living and employment. The psychological impacts were related to the physical issues having effects on mood and social isolation and the VLU dominating all aspects of a person’s life, especially with respect to the potential for embarrassment and shame. The social impacts were visible in relationships with family and friends being described in largely negative terms, with strains on marital and other significant relationships after becoming reliant on the partners for activities of daily living, with odor affecting intimacy and with some people with VLU isolating themselves by canceling events or becoming emotionally isolated through not wanting to discuss their fears. Relationships, however, with health professionals could be positive as professionals engaged closely with those with VLU or quite mechanical with the patients just treated as objects without the patient’s own expertise being acknowledged. What the studies in the review lacked, however, was any consideration of the cultural impacts of having a VLU, especially for people where health is conceptualized as extending beyond the individual.

Our study represents a window into the impact of VLU when considered through the lens of an Indigenous culture, Te Ao Māori, where the conception of healing is more than the closure of a skin lesion. Health and wellbeing are conceptualized by Māori as connected to, and inseparable from, whakapapa (histories and genealogies), te taiao (the environment), wairuatanga (spirituality), whenua (the land), whānau (genetic and chosen family), hinengaro (thoughts and emotions), and tinana (the body; Durie, 1985). The process of engaging with these many layers is through pōwhiri and whanaungatanga (building connections), within which the health professional, patient, and whānau are recognized as whole people located within their cultures, support networks, aspirations, and histories (Pitama et al., 2014). In his foundational work, Durie (1985) asserts that a lack of wellbeing in one or more of these areas results in the whole structure of a person’s wellbeing being vulnerable to collapse. Through this Indigenous lens, the findings of cultural and familial isolation described by our participants are indicative of wholesale ill health that extends far beyond the site of the ulcer to permeate every aspect of the patient and whānau lives. Accounts of choosing to avoid attending culturally important events such as tangihanga (funeral traditions) and other, less formal, whānau gatherings were expressed with grief as a significant loss to the patient. Additionally, there is an associated loss to the wider whānau as the patient’s withdrawal creates a gap in the fabric of cultural connectedness. These additional cultural needs around VLU are not addressed in the literature despite the universal requirement for cultural safety in the delivery of health care.

The findings in our study in many ways also echo the literature about Māori experiences of health care (Graham and Masters-Awatere, 2020). Such literature includes accounts of ongoing colonization and racism, geographical and financial barriers to accessing healthcare, and a disregard for patient expertise. However, this project brings a nuanced approach to understanding the way Māori patients and whānau have experienced and managed VLU in Aotearoa that may resonate with the international community of Indigenous peoples who are similarly located in healthcare systems that disregard and resist their worldviews. Incorporating Indigenous experience into care could help reset the standards for all people with VLU, moving beyond mechanistic performance indicators such as the number of patients with recent ankle-brachial indices, in compression, in preventive hosiery after healing, or in receipt of surgery, to goals that include the experience of care. Such goals could include the recognition of patient expertise in their own care, inclusion of family/whānau in care planning, and acknowledging the imperative of consistency of advice and action within and across the spectrum of care. Education and training too would need to move beyond a focus on the leg. For instance, recognition of the ways in which venous leg ulcers can motivate a person to isolate from family/whānau means that a patient declining to involve whānau should be viewed within the context of the cultural nuances and values that influence how Indigenous communities cope with pain and adversity. Such isolation should not be taken at face value; it needs to be explored so that maintenance or restoration of relationships can be incorporated as part of a holistic approach to healing.

### Strengths and limitations

The study strengths include being the first study to investigate the experience of VLU in an Indigenous population, using a pūrākau (storied) approach so that participants could create their own narratives, employing a Māori interviewer who engaged with the participants using practices consistent with the culture of Māori, and including whānau in the conversations. However, only two participants chose to include their whānau in the interviews and this points to a possible limitation of the study, namely the relative absence of whānau whānui (wider family) voice in the narratives. A further limitation is that recruitment was drawn from a convenience sample of former trial participants. Such a sample might not be representative of the Māori who were not eligible to participate in the trials. However, the trials used pragmatic designs to keep the inclusion criteria as open as possible and the number of Māori recruited (62 out of 362) was the same as the population fraction for Māori in the total population (17%).

## Conclusion

Acknowledging and integrating Indigenous experiences into care would reset service provision for all patients with VLU. Recognizing and respecting patients’ expertise in their condition, the inclusion of whānau in care planning and provision, while providing consistency in advice and resource access, would enhance venous ulcer management for every patient. Training in VLU care needs to move beyond a focus on the leg and toward a more holistic approach that refocuses on the whole person and those connected to them. Future investigations into the experiences of patients with VLU should recognize and explore additive effects of the participants’ culture. Integration of cultural aspects into care practices will ensure a more comprehensive understanding of patient and whānau needs, paving a way for culturally safe and effective care practices.
